# Improvement of image resolution by combining enhanced confocal microscopy and quantum dot triexciton imaging

**DOI:** 10.1002/2211-5463.13246

**Published:** 2021-07-26

**Authors:** Simon Hennig, Dietmar J. Manstein

**Affiliations:** ^1^ Institute for Biophysical Chemistry OE4350 Hannover Medical School Fritz‐Hartmann‐Centre for Medical Research Germany; ^2^ Division for Structural Biochemistry, OE8830, Hannover Medical School Hannover Germany; ^3^ RESiST Cluster of Excellence 2155 Medizinische Hochschule Hannover Germany

**Keywords:** quantum dot triexciton imaging, Airyscan, STELLARIS, 3D confocal imaging, nanoscopy, super‐resolution fluorescence imaging, quantum dots

## Abstract

Super‐resolution fluorescence imaging provides critically improved information about the composition, organization, and dynamics of subcellular structures. Quantum dot triexciton imaging (QDTI) has been introduced as an easy‐to‐use sub‐diffraction imaging method that achieves an almost 2‐fold improvement in resolution when used with conventional confocal microscopes. Here, we report an overall 3‐fold increase in lateral and axial resolution compared to conventional confocal microscopes by combining QDTI with state‐of‐the‐art commercial laser scanning microscope systems.

## Graphical abstract

We report an overall 3‐fold increase in lateral and axial image resolution for confocal fluorescence microscopy by combining quantum dot triexciton imaging with state‐of‐the‐art commercial laser scanning microscope systems. Based on quantum dots as fluorescent labels, we present an easy‐to‐use super‐resolution method. A single confocal scan with minimal image post‐processing is sufficient to produce a sub‐diffraction image with an image resolution of approx. 80 nm lateral and 200 nm axial.

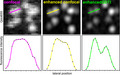

**Abbreviations**
QDTI: quantum dot triexciton imaging
MX: monoexciton
TX: triexciton
QD: quantum dot
CW: continuous wave
QdSe: cadmium selenide
LSM: laser scanning microscope
PVA: polyvinyl alcohol
AU: airy unit
SNR: signal‐to‐noise ratio

Access to imaging techniques that resolve structures at the molecular level is now widely available [1, 2]. However, imaging below the diffraction limit is still associated with some pitfalls and can be difficult to apply to a specific problem. Quantum dot triexciton imaging (QDTI) [3, 4, 5] is an easy‐to‐use, high‐resolution confocal imaging method based on the generation and detection of a tri‐excitonic (TX) state by successive absorption of three photons in quantum dots (QDs). QD655 are cadmium selenide (CdSe) QDs which, in addition to their use in conventional fluorescence microscopy applications with detection of their mono‐excitonic (MX) emissions, allow the generation and sensitive detection of higher excitonic states. These higher excitonic states can be readily generated using pulsed or continuous wave (CW) lasers in the range between 350 and 488 nm. The unconventional recombination of triple excitons via the p–p recombination channel [6] produces a characteristic blue‐shifted TX emission line at approx. 615 nm, which is readily separated spectrally from the common s–s recombination channel generating the MX emission line at 655 nm [7, 8]. The detection of TX instead of MX emission leads to increases in lateral and axial resolution adding—besides a high quantum yield, which is intrinsic to quantum dots [9]—to the attractiveness of QD655 as a probe for fluorescence imaging.

To test the extent to which the application of QDTI with state‐of‐the‐art commercial laser scanning microscope (LSM) systems improves the spatial resolution in confocal imaging, we used QDTI in combination with Zeiss Airyscan 2 and Leica STELLARIS 8 systems. Using different technical approaches, both systems are capable of exceeding the diffraction limit of a conventional confocal microscope by a factor of up to 2. The Airyscan 2 uses an area detector with concentrically arranged detection elements combined with an open pinhole to collect emitted photons with increased efficiency and sensitivity. Photons collected in the outer rings are re‐assigned to the centrally located detector element and in combination with ‘Airy filtering’, which corresponds to the application of a linear Wiener noise filter, generates the final super‐resolved image [10, 11]. The Leica STELLARIS 8 LIGHTNING uses Power HyD detectors for confocal imaging. The high sensitivity of the HyD detectors allows for imaging with a small physical pinhole and thus natively increases the resolution already in confocal scanning [12]. Subsequent application of efficient and fast adaptive deconvolution (LIGHTNING) produces the final super‐resolved confocal image.

When we use QDTI with these state‐of‐the‐art commercial LSM systems, an additional 1.5‐fold resolution improvement is achieved, resulting in an overall 3‐fold resolution improvement compared to conventional confocal imaging. The combined approach, which we refer to as ‘enhanced QDTI’ (eQDTI), allows imaging down to a lateral and axial resolution of 81 nm and 210 nm, respectively. The method is easy to use. A single confocal scan is required to generate the super‐resolved image. Post‐processing is limited to the use of ‘Airy Filtering’ (Zeiss) and ‘LIGHTNING’ (Leica), which generate results almost instantaneously. Since eQDTI is based solely on the physical effect of triple exciton generation, it is compatible with conventional buffers or mounting media and requires no elaborate or special sample preparation.

## Methods

Experiments were performed using a Zeiss LSM980 with Airyscan 2 and 34 channel QUASAR detection unit. The principle of the Airyscan detector can be found elsewhere [9, 10]. The 405 nm diode laser of the microscope was employed for excitation of QDs. Since the Zeiss microscopy software uses only percentage scales to set the laser power, the excitation intensity was measured directly at the front lens of the objective to determine the absolute values in watts. For our system, a nearly linear dependence from 2.5 µW at 0.2 to 1.06 mW at 100% between the two scales was found. Specifically, the applied and measured values of 0.1, 1, 10, 50, and 100% correspond to intensities of 1.5 µW (0.1%), 11.22 µW (1%), 107.5 µW (10%), 532.7 µW (50%), and 1063 µW (100%). Confocal MX imaging was carried out with the standard 63×, 1.46NA oil immersion objective, a setting of 70 nm/pixel, a detection window of 630–700 nm, amplification gain 650 V, and 2‐fold sampling. The pinhole was set to 1 AU. Airyscan imaging was carried out with 30 nm/pixel using 605–705 (MX) and 525–585 nm (TX) filters. Pixel dwell times were set to 37.66–68.23 µs for Airyscan MX imaging and 78.2–135.3 µs for Airyscan TX imaging with 2‐fold sampling. Detector gain was set to 750 V and 950 V, respectively. 3D imaging was carried out with 210 nm/plane for confocal and 120 nm/plane for Airyscan imaging. To avoid image artifacts and to ensure a consistent correction of the images in post‐processing with ZenBlue, we used a constant value of 5 for the Airy filter and deactivated the automatic settings. Interpolation was switched off during all image processing steps. Laser intensities were set to 0.4% for MX imaging and 4% for TX imaging in cell images.

STELLARIS images were captured at an STELLARIS 8 system. The 405 nm diode laser was selected to excite quantum dots. Confocal imaging was performed with the 63×, 1.2NA water immersion objective. Hybrid detectors at detection wavelength of 525 – 605 nm (TX channel) and 615–705 nm (MX channel) were employed to detect fluorescence light. The pinhole was set to 0.6 Airy Units. Scanning was performed, using 40 nm/px with 12 µs/px and 2‐fold sampling. Post‐processing was performed using the LAS X LIGHTNING [13]

FWHM measurements were performed in PBS buffer, which contained 1 mm 2‐mercaptoethanol to suppress QD655 blinking [14]. Comparison of MX and TX emission was performed by increasing the excitation intensity in 0.2% steps from 0.4 to 2% for MX detection and in 2.5 or 5% steps from 2.5 to 35% for TX detection. Images were processed and analyzed using FIJI [15]. Normalization of images from the three different channels was achieved by using ‘Contrast Enhancer’ (Process > Enhance Contrast) with ‘normalization’. LUTs and greyscales are linear representations of the raw or normalized values. A549 and U‐2 OS cells (Sigma‐Aldrich, USA) were cultured in DMEM/F‐12 medium (Merck KGaA, Darmstadt, Germany). Cells were seeded in LabTek II chamber slides (NUNC) 48 h prior to measurements. The protocol for fixation and immunolabeling of microtubules with QD655 (Thermo Fisher Scientific, Waltham, MA, USA, #Q‐11021MP) can be found elsewhere [3].

Spectral measurements were performed using the QUASAR detector of the Zeiss Airyscan 2 (Fig. S1). The detector provides a spectral resolution of ˜ 9 nm. Spectra were obtained from a single QD655 quantum dot spincoated onto a coverslip and embedded in PVA. The QDot was excited at 405 nm and pixel dwell times of 70 µs were used. Spectra were acquired with illumination settings of 0.2, 1, 5, and 10 percent corresponding to the range from ˜ 2.5 to 120 µW, on our system. The spectrally encoded images were further processed in Fiji and then analyzed using Origin Pro. Filter settings for the Airyscan 2 detector were derived accordingly (Fig. S1D colored areas in the background). A blue‐shifted shoulder is observed in the spectra with increasing illumination intensity. Analysis using a double Gaussian distribution fit identifies emission peaks at 659.37 ± 0.7 nm and 625.86 ± 1.8 nm. These values differ slightly from the reported emission peaks at 655 nm and 619 nm [8]. We attribute the observed spectral differences to environmental changes such as temperature, hydration, and embedding of the QDots.

## Results and discussion

To determine the resolving power of eQDTI, we prepared surfaces that are sparsely decorated with QD655 quantum dots. As the full width at half maximum (FWHM) detected from emitters depends strongly on the applied excitation intensity [3], we determined the changes in apparent FWHM for individual quantum dots as a function of the excitation intensity. To demonstrate the suitability and applicability of eQDTI, we furthermore recorded the filamentous network of microtubule‐based structures in A549 and U‐2 OS cells using different image acquisition modalities. Scanning with the previously determined optimal imaging conditions for each detection channel, we subsequently generated images of the same region of interest.

**Fig. 1:**
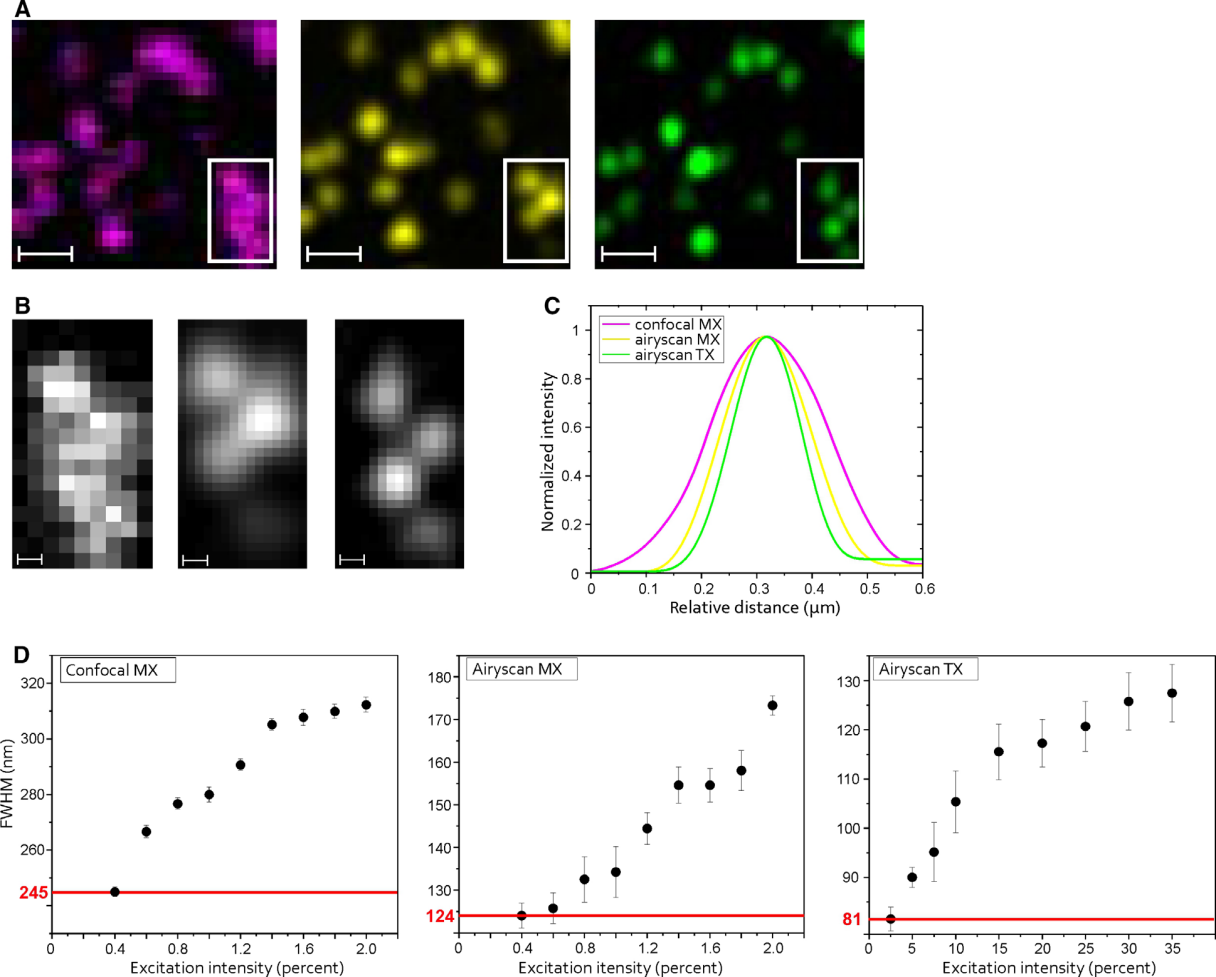
Characterization of eQDTI imaging using sparsely decorated QD655 surfaces. (A) The same region of interest showing a surface area sparsely decorated with QD655 quantum dots was imaged using the Confocal MX (magenta), the Airyscan MX (yellow), and Airyscan TX (green) channel. Scale bar, 500 nm. (B) Expanded areas from a (rectangles) showing a cluster of quantum dots in the Confocal MX (left), Airyscan MX (middle), and Airyscan TX (right) channel. Scale bar, 100 nm. (C) Normalized point spread function of a representative fluorescent spot, showing the intensity distribution in the three emission channels. (D) Plots showing the lateral FWHM obtained from a single quantum dot as function of the excitation intensity. All three emission channels show a decreasing FWHM with decreasing excitation intensity. The Confocal MX channel shows a limit at 245 nm, the Airyscan MX channel at 124 nm, and the Airyscan TX channel at 81 nm, showing a 2‐fold lateral resolution enhancement from Confocal MX to Airyscan MX and 3‐fold lateral resolution enhancement from Confocal MX to Airyscan TX. Error bars represent the standard error.

Fig. 1A shows a representative region of interest that was sequentially imaged using Confocal MX, Airyscan MX, and Airyscan TX detection at the Airyscan 2 microscope. The resulting point spread functions and the dependence of the lateral FWHM on excitation intensity illustrate the up to 3‐fold improvement in lateral resolution achieved by applying the eQDTI approach (Fig. 1B, C and D). The limitation of the achieved resolution in TX imaging strongly correlates with the excitation intensity in combination with the pixel dwell time and consequently the signal‐to‐noise ratio (SNR). When comparing the probabilities of generating and detecting TX emissions via the p–p recombination channel and generating and detecting a MX state in QDs, the probability is much lower in the TX case. This can be compensated for by a higher pixel dwell time, but has its limitations. Therefore, when the excitation intensity is reduced, the TX SNR decreases faster than the MX SNR, limiting the detection of TX signals in our experiments at about 2.5% excitation intensity instead of 0.2% in the MX emission case. The respective limits in our experiments are marked by red lines (Fig. 1D). Since the QDTI method improves the resolution in all three dimensions, the eQDTI approach leads also to a reduction of the axial FWHM. 3D xz visualization of a sparsely decorated QD655 surface indicates the axial resolution improvement for the Airyscan TX channel, compared to the Confocal MX and Airyscan MX channels (Fig. S2). Our estimate of an axial resolution of about 210 nm achieved by eQDTI is based on the reported axial resolution of 350 nm for z‐stacks recorded in Airyscan super‐resolution mode and a further 1.7‐fold gain by QDTI [3]. The improved axial resolution results simultaneously in a reduced image background. This is most obvious in images with crowded and densely organized structures, as shown in Fig. S3.

**Fig. 2:**
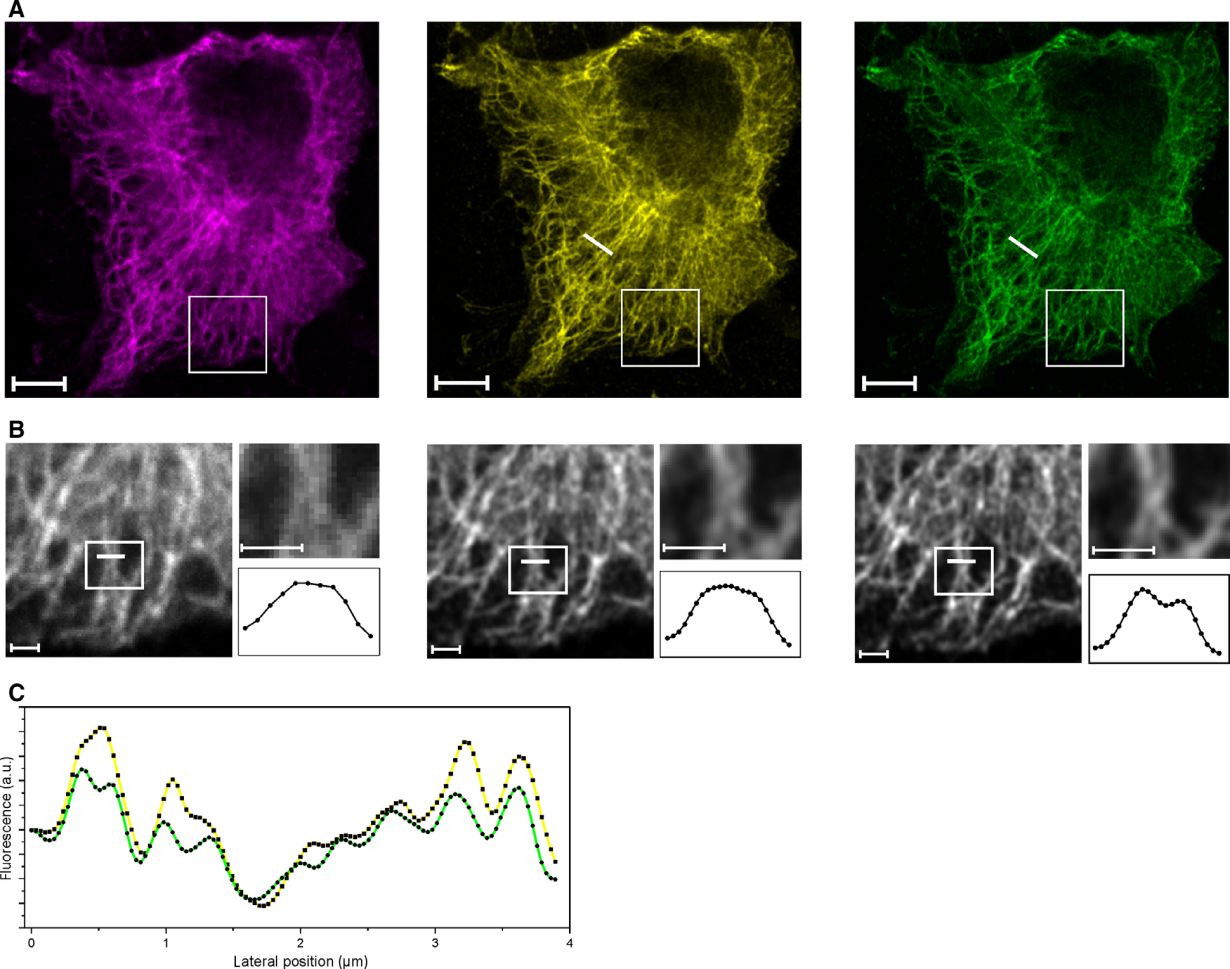
eQDTI‐mediated improvements in the resolution of cytoskeletal structures at the Zeiss Airyscan 2. (A) Region of interest showing a fixed A549 cell, immunolabeled for microtubules with QD655 quantum dots and imaged subsequently by utilizing the Confocal MX (magenta), Airyscan MX (yellow), and Airyscan TX (green) emission channel. Scale bar, 5 µm. (B) Insets, taken from a showing the detailed distribution of filamentous structures in the respective channels. Fine details can be observed in the Airyscan TX channel, which are not observable in the Confocal MX or Airyscan MX channel (insets). Nearby structures are resolved with greater detail (white lines and associated intensity profiles). Scale bars, 1 µm. (C) Color‐coded intensity profiles (Airyscan Mx, yellow and Airyscan TX, green) taken from cross section in a (white lines).

**Fig. 3:**
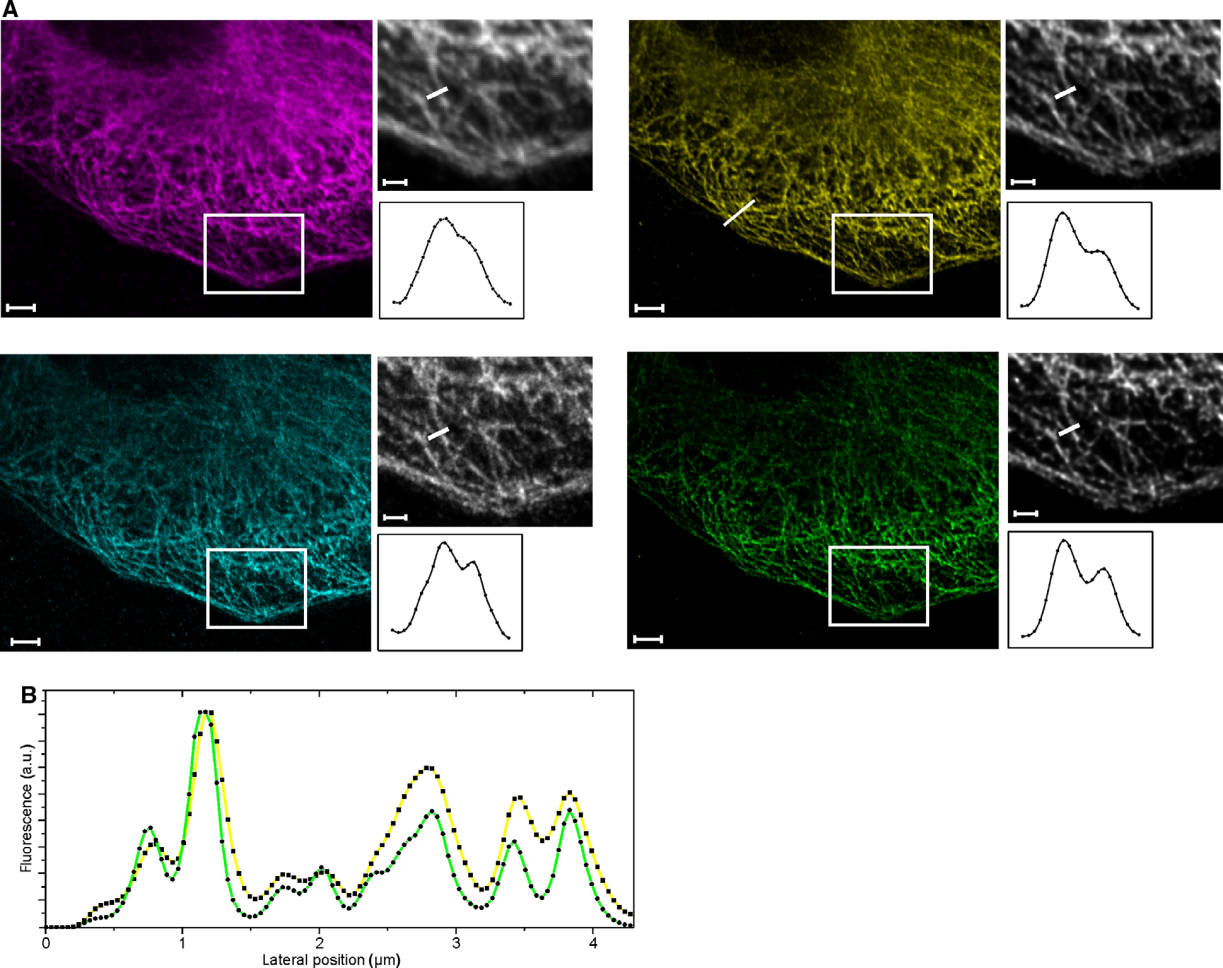
eQDTI‐mediated improvements in the resolution of cytoskeletal structures at the Leica STELLARIS 8. (A) Region of interest showing a fixed U2OS cell, immunolabeled for microtubules with QD655 quantum dots, and imaged subsequently by utilizing the Confocal MX (magenta) and Confocal TX (cyan) emission channel. The LIGHTNING MX (yellow) and LIGHTNING TX image (green) was processed from the Confocal MX and TX emission channel respectively. Insets, taken from original images showing the detailed distribution of filamentous structures in the respective channels. Scale bar, 5 µm; insets, 1 µm. (B) Color‐coded intensity profiles taken from a cross section (see LIGHTNING MX image in a. Opposed are the and the LIGHTNING MX in yellow and LIGHTNING TX in green channels.

Figs 2 and 3 show the structures of microtubule filaments in A549 and U‐2 OS cells visualized by immunolabeling of beta‐tubulin with QD655‐labeled antibodies. To visualize the impact of QDTI, we recorded images of microtubule structures using the Airyscan 2 and STELLARIS 8 systems in different imaging modes. For both systems, we performed conventional confocal MX imaging as well as high‐resolution confocal imaging employing Airyscan in combination with Airy filtering (Carl Zeiss Microscopy, Oberkochen, Germany) and HyD detectors in combination with the LIGHTNING algorithm (Leica Microsystems, Wetzlar, Germany). Fig. 2 presents images taken at the Airyscan 2. Besides the confocal image, the Airyscan MX and the Airyscan TX images are shown (Fig. 2A). Comparing the high‐resolution images with each other reveals finer mapped structures for the TX emission compared to the MX emission images from the Airy‐detector (Fig. 2B with insets). Furthermore, the Airyscan TX detection shows details, which are blurred in the Airyscan MX channel (Fig. 2B line profiles). An intensity line profile through a representative area of the cell shows the consistently higher resolved structures in the Airyscan TX image (Fig. 2C). Figure 3 shows images taken with the STELLARIS 8. The confocal MX and TX images with their respective LIGHTNING pendants are depicted (Fig. 3A). As mentioned previously, the confocal images shown here provide an already increased resolution compared to conventional confocal systems as sensitive detectors and a successively smaller pinhole can be leveraged by this system. This has to be considered, when comparing the relative resolution enhancement. The LIGHTNING‐processed images show an improved image quality as well as a reduced background, which is directly visible when comparing confocal with the processed images (Fig. 3A). When comparing the LIGHTNING MX and TX images, the TX channel shows greater detail and finer structures, compared to the MX channel (Fig. 3A insets and line profiles). In Fig. 3B, normalized intensity line profiles of the high‐resolution LIGHTNING MX and TX from a region of interest are depicted. The LIGHTNING TX line profile shows a clearly more detailed intensity course, compared to the respective MX counterpart.

## Conclusions

In summary, we describe a straightforward and readily applicable confocal imaging technique based on the generation of three exciton states in quantum dots in combination with latest generation of confocal microscopes which is capable to resolve fluorescence signals with a precision of 81 nm laterally and around 210 nm axially. To generate the super‐resolved image, labeling the structures of interest with QD655 quantum dots in combination with a single scan to detect the blue‐shifted TX emission followed by the application of the appropriate straightforward post‐processing methods is sufficient.

## Conflict of interest

The authors declare no conflict of interest.

## Author contributions

SH designed the project, performed imaging and image analysis. SH and DJM interpreted results and wrote the manuscript.

## Supporting information


**Fig. S1**. Fluorescence spectra of a single QD655, taken at different illumination intensities. (A–D) acquired fluorescence spectra (black squares) with spectral sampling of 9 nm taken with the spectral detector at the Zeiss Airyscan 2 microscope at an illumination wavelength of 405 nm. Illumination intensities: (A) 0.2% (2.52 μW), (B) 1% (11.22 μW), (C) 5% (54.25 μW), (D) 10% (107.5 μW). Note the increasing intensity of the TX emission peak, indicated by the unsymmetrical emission spectra. Solid lines: double gaussian distribution fits of the emission spectra. The fits reveal two emission peaks in each acquired spectrum. The TX emission peak (green) located at 625.86 ± 1.8 nm and the MX peak (red), which was located at 659.37 ± 0.73 nm. The blue solid line shows the cumulative fit. (D) Colored regions in the background of the spectrum represent the emission filter settings used for the Airyscan detector. Green: TX emission filter, red: MX emission filter. (E) normalized emission spectra at 0.2% (black) and 10% (blue) excitation power. Assuming a negligible TX emission at 0.2% excitation, subtraction of spectra reveals the extracted TX emission in the difference spectrum with a maximum at 627 nm (see inset with gaussian fit).
**Fig. S2**. Investigation of Axial Point Spread Functions of QD655 emitters. (A) 3D representation of a glass surface sparsely decorated with QD655 emitters. Channels from left to right: Confocal MX, Airscan MX, and Airyscan TX. Images were generated, using the 3D image view of the ZenBlue software. Image intensities were matched using the min/max function and a setting of 13% for the high‐pass intensity filter. (B) Maximum intensity projections of a single QD655 emitter recorded from left to right in the Confocal MX, Airyscan MX and Airyscan TX emission channels. Intensities were normalized as described in the Methods section. Scale bar, 500 nm.
**Fig. S3**. Fluorescence background reduction induced by eQDTI. (A) Region of interest showing a dense network of QD655‐labeled microtubules imaged in the Airyscan MX channel (left) and Airyscan TX channel (right). Scale bar, 2 μm. (B) Normalized intensity profiles, illustrating the extent to which the background intensity in the Airyscan TX channel is reduced (double headed arrow). The enhanced lateral resolution of the Airyscan TX channel resolves finer details (blue circle).

## Data Availability

The data that support the findings of this study are available in Supplementary Information. Raw data are available from the corresponding author, SH upon reasonable request.
