# Supplementary material for: Improvement of image resolution by combining enhanced confocal microscopy and quantum dot triexciton imaging
Source: FEBS Open Bio. 2021 Jul 26;11(12):3324–30. doi: 10.1002/2211-5463.13246 (PMC8634860; doi:10.1002/2211-5463.13246)
Supplement: Supplementary file 1 — Fig. S1. Fluorescence spectra of a single QD655, taken at different illumination intensities. (A–D) acquired fluorescence spectra (black squares) with spectral sampling of 9 nm taken with the spectral detector at the Zeiss Airyscan 2 microscope at an illumination wavelength of 405 nm. Illumination intensities: (A) 0.2% (2.52 μW), (B) 1% (11.22 μW), (C) 5% (54.25 μW), (D) 10% (107.5 μW). Note the increasing intensity of the TX emission peak, indicated by the unsymmetrical emission spectra. Solid lines: double gaussian distribution fits of the emission spectra. The fits reveal two emission peaks in each acquired spectrum. The TX emission peak (green) located at 625.86 ± 1.8 nm and the MX peak (red), which was located at 659.37 ± 0.73 nm. The blue solid line shows the cumulative fit. (D) Colored regions in the background of the spectrum represent the emission filter settings used for the Airyscan detector. Green: TX emission filter, red: MX emission filter. (E) normalized emission spectra at 0.2% (black) and 10% (blue) excitation power. Assuming a negligible TX emission at 0.2% excitation, subtraction of spectra reveals the extracted TX emission in the difference spectrum with a maximum at 627 nm (see inset with gaussian fit). Fig. S2. Investigation of Axial Point Spread Functions of QD655 emitters. (A) 3D representation of a glass surface sparsely decorated with QD655 emitters. Channels from left to right: Confocal MX, Airscan MX, and Airyscan TX. Images were generated, using the 3D image view of the ZenBlue software. Image intensities were matched using the min/max function and a setting of 13% for the high‐pass intensity filter. (B) Maximum intensity projections of a single QD655 emitter recorded from left to right in the Confocal MX, Airyscan MX and Airyscan TX emission channels. Intensities were normalized as described in the Methods section. Scale bar, 500 nm. Fig. S3. Fluorescence background reduction induced by eQDTI. (A) Region of interest showing a dense netw [file FEB4-11-3324-s001.pdf]

Supplementary information

Improvement of image resolution by combining enhanced confocal microscopy and quantum dot triexciton imaging

Simon Hennig[1] and Dietmar J. Manstein[1,2,3]

[1] Institute for Biophysical Chemistry, OE4350, Hannover Medical School, Fritz-Hartmann-Centre for Medical Research, 30625 Hannover, Germany

[2] Division for Structural Biochemistry, OE8830, Carl-Neuberg-Str.1, 30625 Hannover, Germany

[3] RESiST, Cluster of Excellence 2155, Medizinische Hochschule Hannover, 30625 Hannover, Germany

Supplementary Figures:

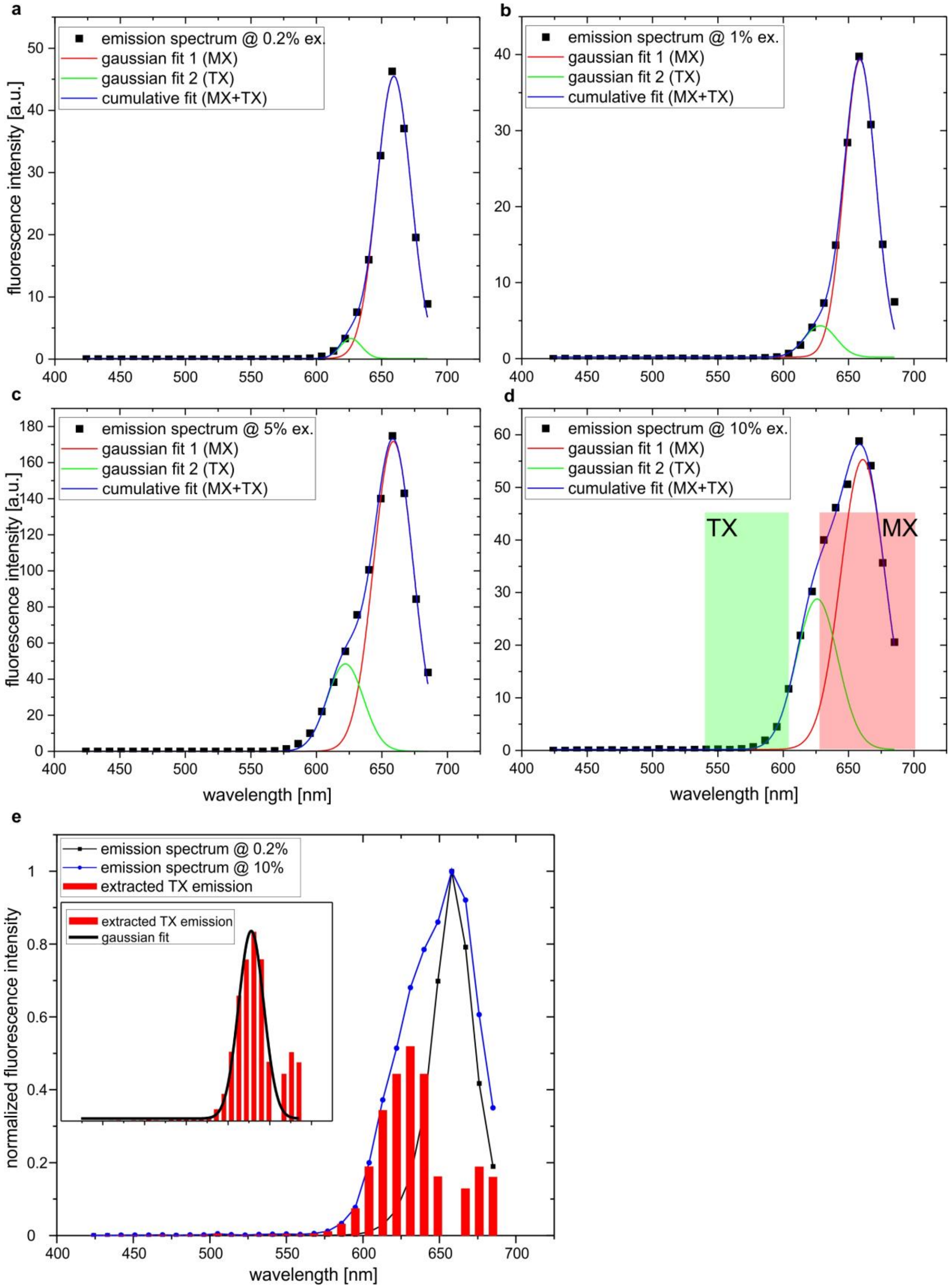

Fig. S1 Fluorescence spectra of a single QD655, taken at different illumination intensities. a-d: acquired fluorescence spectra (black squares) with spectral sampling of 9 nm taken with the spectral detector at the Zeiss Airyscan 2 microscope at an illumination wavelength of 405 nm. Illumination intensities: **a**: 0.2% (2.52 µW), **b**: 1% (11.22 µW), **c**: 5% (54.25 µW), **d**: 10% (107.5 µW). Note the increasing intensity of the TX emission peak, indicated by the unsymmetrical emission spectra. Solid lines: double gaussian distribution fits of the emission spectra. The fits reveal two emission peaks in each acquired spectrum. The TX emission peak (green) located at 625.86 ±1.8 nm. and the MX peak (red), which was located at 659.37 ±0.73 nm. The blue solid line shows the cumulative fit. **d**: Colored regions in the background of the spectrum represent the emission filter settings used for the Airyscan detector. Green: TX emission filter, red: MX emission filter. **e**: normalized emission spectra at 0.2% (black) and 10% (blue) excitation power. Assuming a negligible TX emission at 0.2% excitation, subtraction of spectra reveals the extracted TX emission in the difference spectrum with a maximum at 627 nm (see inset with gaussian fit).

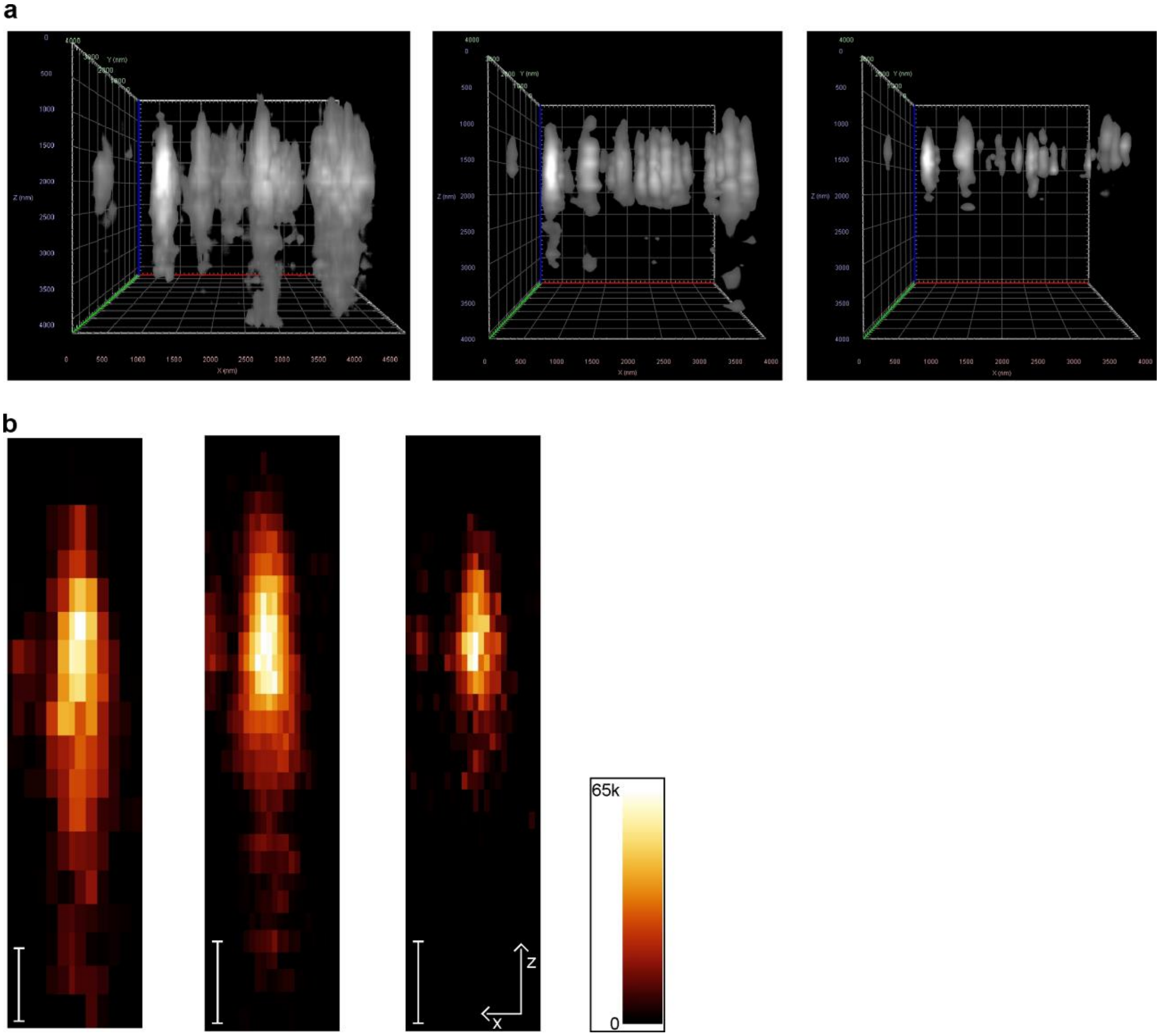

Fig. S2 Investigation of Axial Point Spread Functions of QD655 emitters. a, 3D representation of a glass surface sparsely decorated with QD655 emitters. Channels from left to right: Confocal MX, Airscan MX, and Airyscan TX. Images were generated, using the 3D image view of the ZenBlue software. Image intensities were matched using the min/max function and a setting of 13% for the high-pass intensity filter. **b** Maximum intensity projections of a single QD655 emitter recorded from left to right in the Confocal MX, Airyscan MX and Airyscan TX emission channels. Intensities were normalized as described in the Methods section. Scale bar, 500 nm.

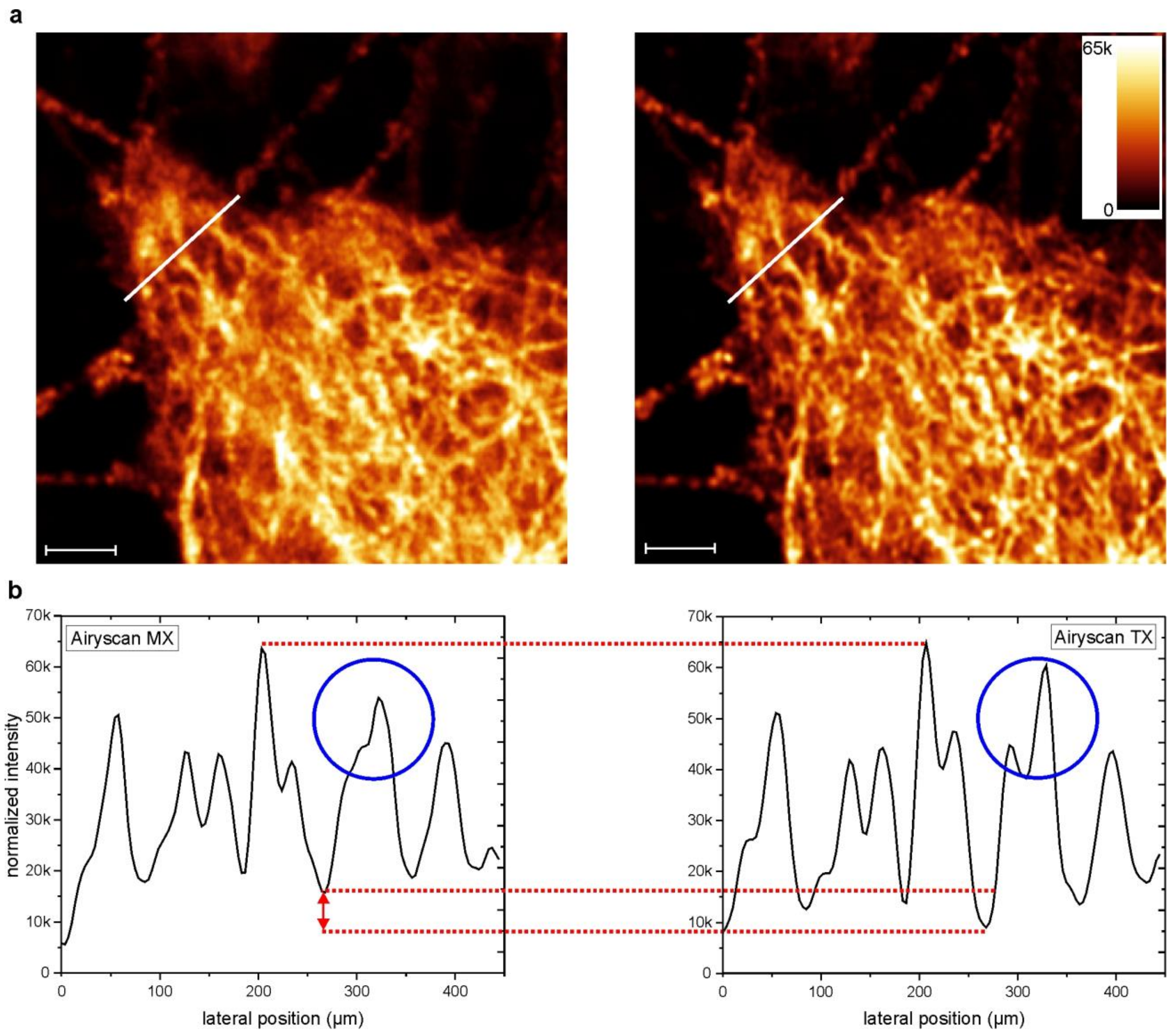

Fig. S3 Fluorescence background reduction induced by eQDTI. a, Region of interest showing a dense network of QD655-labeled microtubules imaged in the Airyscan MX channel (left) and Airyscan TX channel (right). Scale bar, 2 µm. **b**, Normalized intensity profiles, illustrating the extent to which the background intensity in the Airyscan TX channel is reduced (double headed arrow). The enhanced lateral resolution of the Airyscan TX channel resolves finer details (blue circle).
